# Supplementary material for: Ambrosia beetle invasions are structured by inbreeding, intraspecific hybridization, and bridgeheads
Source: Mol Biol Evol. 2026 Sep 8;43(9):msag231. doi: 10.1093/molbev/msag231 (PMC13584198; doi:10.1093/molbev/msag231)
Supplement: Supplementary file 1 [file msag231_supplementary_data.zip › Schmidt Supp Figs S1-S4 PSHB.pdf]

Fig. S1. PCAs of SHB.

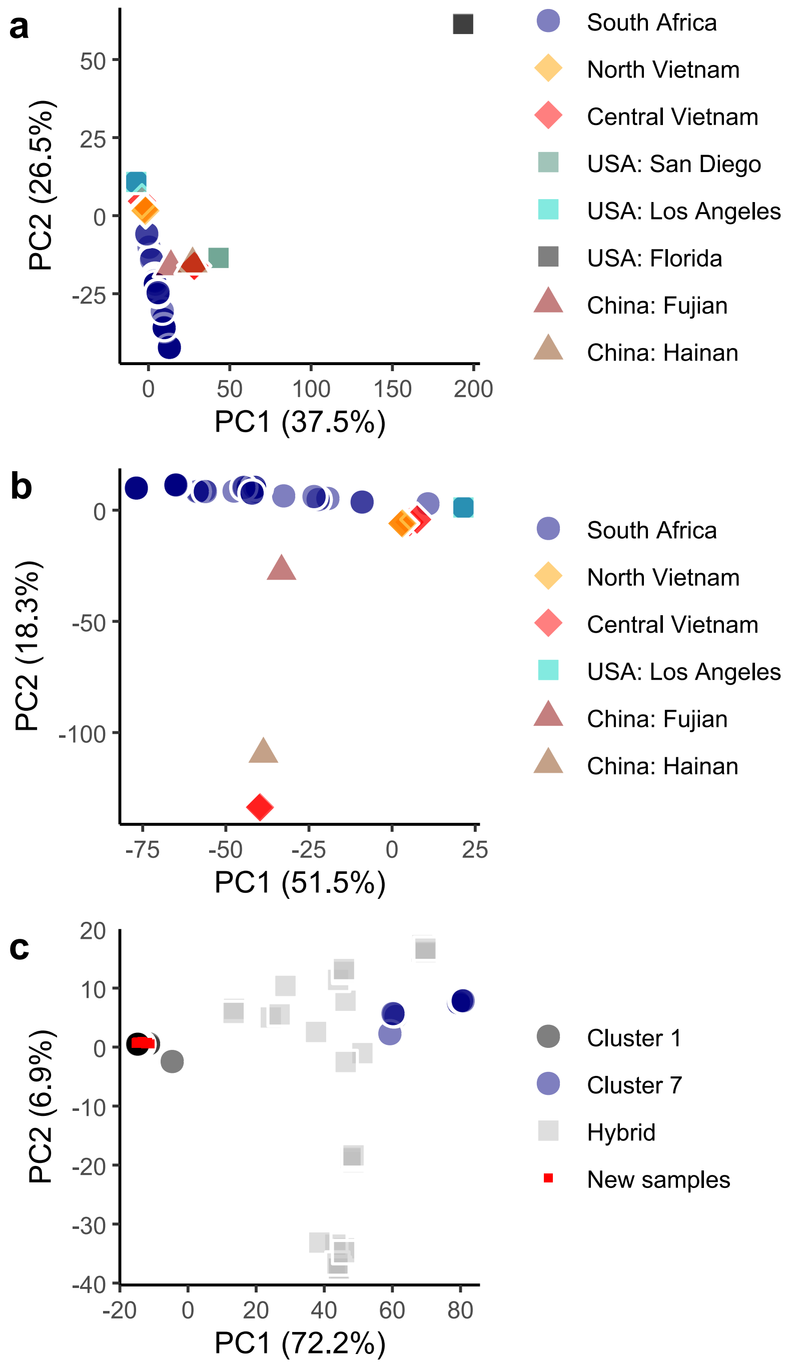

(a) PCA of all SHB populations. (b) PCA of all PSHB populations. (c) PCA of South African PSHB samples, including the 59 additional samples from 26 locations (Fig. 2c).

Fig. S2. Heatmap showing PSHB genotypes across the first six chromosomes (I–VI).

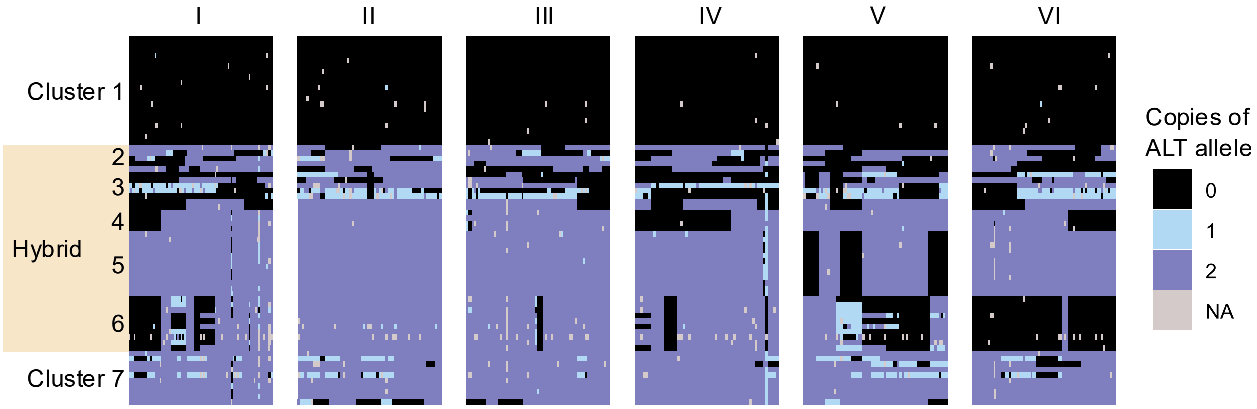

Individuals are sorted by cluster on y-axis. SNP genotypes are sorted by position on x-axis.

Fig. S3. Proportion of alleles identical to the reference genome.

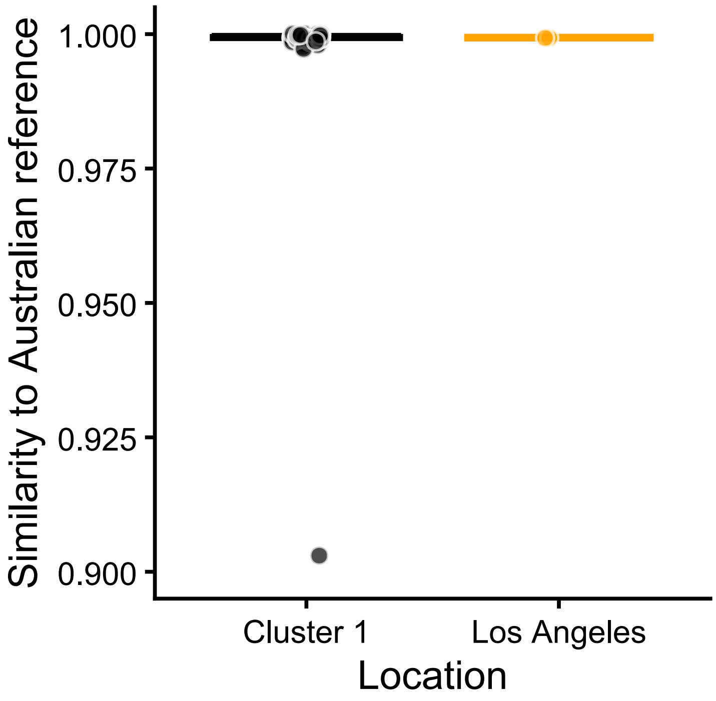

Alleles are at 3060 SNPs called within South African Cluster 1 and 7, and hybrid clusters. Thirteen Cluster 1 samples had 100% similarity, while one sample was admixed. Each Los Angeles sample had four non-reference alleles.

Fig. S4. Distribution of Cluster 1 mtDNA haplotypes in South Africa

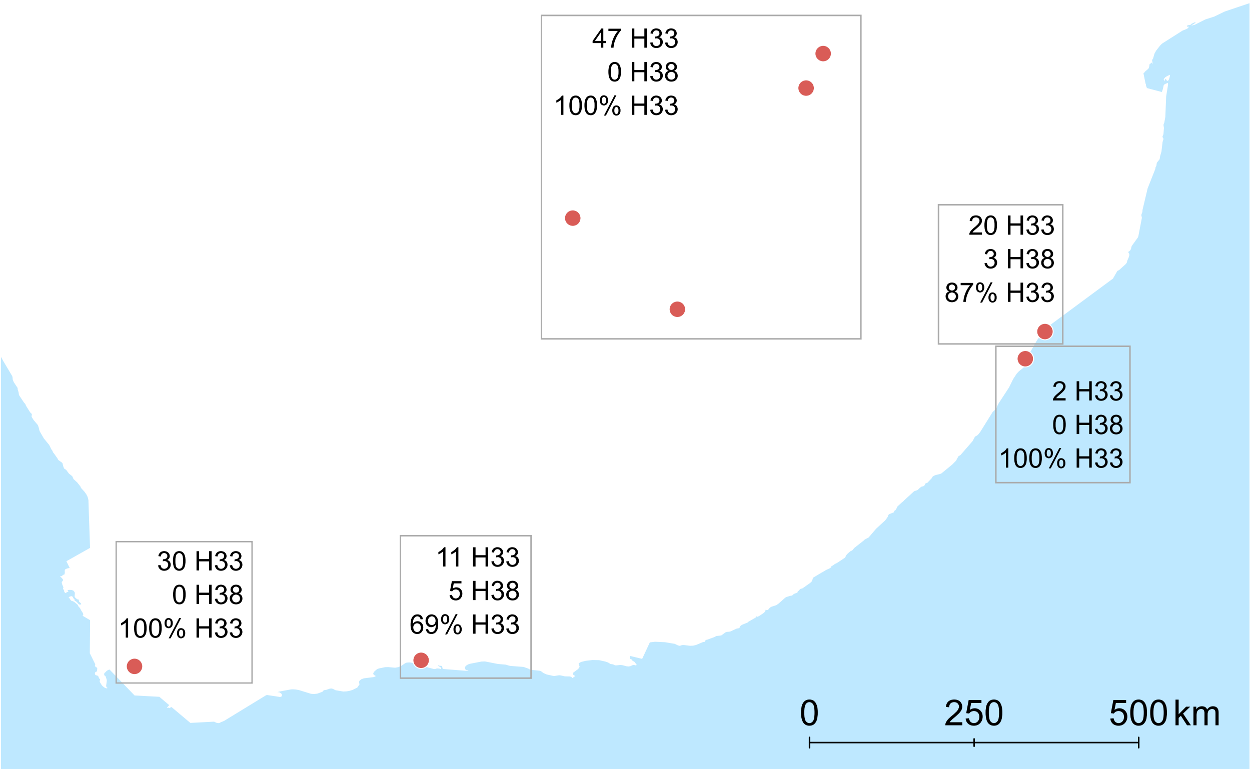

Symbols indicate broad sampling locations. Boxes summarise the local haplotype proportions. Haplotypes of Clusters 2–7 are in Table S1.
